# Do Religiosity and Spirituality Differ in Their Relationship with Crystallized Intelligence? Evidence from the General Social Survey

**DOI:** 10.3390/jintelligence12070065

**Published:** 2024-07-07

**Authors:** Florian Dürlinger, Thomas Goetz, Jakob Pietschnig

**Affiliations:** Department of Developmental and Educational Psychology, Faculty of Psychology, University of Vienna, Liebiggasse 5, 1010 Wien, Austria; thomas.goetz@univie.ac.at (T.G.); jakob.pietschnig@univie.ac.at (J.P.)

**Keywords:** religiosity, spirituality, intelligence, time-trends, GSS

## Abstract

Negative associations of religiosity and intelligence are well established in psychological research. However, past studies have shown a substantial heterogeneity in reported effect strengths. Causes that may be able to explain the identified inconsistencies pertain to differing religiosity measurement modalities, participant ages, or possibly cohort effects due to changing societal values in terms of being religious. Moreover, little is known about intelligence associations with the religiosity-related yet distinct construct of spirituality. Here, we provide evidence for religiosity and crystallized intelligence, as well as spirituality and crystallized intelligence associations, in 14 cohorts from 1988 to 2022 (*N* = 35,093) in the General Social Survey data by means of primary data analyses and meta-analytical approaches. As expected, religiosity was non-trivially negatively associated (*r* = −0.13, *p* < .001), but spirituality showed no meaningful association with crystallized intelligence (*r* = 0.03, *p* < .001). Our results broadly generalized across age groups, cohorts, and analytical approaches, thus suggesting that religiosity and intelligence may possibly be functionally equivalent to a certain extent whilst spirituality represents a distinct construct that is not functionally equivalent.

## 1. Introduction

### 1.1. Religiosity and Intelligence

Research about religiosity and intelligence associations goes back almost a century by now, with the first formal empirical studies published in 1928 (Howells 1928; Sinclair 1928). Since then, a plethora of corresponding reports have supported negative associations between religiosity and intelligence. Meta-analytical examinations have corroborated the robustness of this association (Zuckerman et al. 2013, 2020), suggesting that this link generalizes across various moderators in terms of effect direction (e.g., in regard to nationality or culture; e.g., Cribari-Neto and Souza 2013; Lynn et al. 2009) but appears to differ in terms of effect strength (i.e., stronger cognitive ability associations with religious beliefs vs. involvement; Dürlinger and Pietschnig 2022). A participant’s age has been argued to represent another potential cause of effect size variation as in older age, religiosity may have protective effects against cognitive decline (e.g., Hill et al. 2020). However, recent evidence does not support this idea (Dürlinger et al. 2023).

It has been suggested that the negative association between religiosity and intelligence may be rooted in one or a combined effect of three broad causal categories (for an overview, see Zuckerman et al. 2013). First, more intelligent persons tend to prefer an analytic cognitive style over an intuitive cognitive style (Frederick 2005). Adopting an analytic thinking style can lead to a decrease in religiosity (Gervais and Norenzayan 2012). Second, religiosity is believed to fulfill psychological needs and desires (Sedikides 2010), which, in turn, could be obtained by intelligence as well. For instance, both religion (McCullough and Willoughby 2009) and intelligence (Shamosh and Gray 2008) are positively associated with self-regulation and self-control, which, in turn, could lead to positive outcomes, including well-being and academic achievement (McCullough and Willoughby 2009). Furthermore, both religiosity (Sedikides and Gebauer 2010) and intelligence (Miller and Lachman 2000; Schermer and Vernon 2010) have been linked to higher beliefs in compensatory control and self-enhancement. In addition, both religiosity (Epley et al. 2008) and intelligence (Herrnstein and Murray 1994) can help in lowering feelings of loneliness (e.g., negative associations between intelligence and divorce likelihood; Holley et al. 2006). Such common characteristics of religion and intelligence support the notion that these constructs are functionally equivalent to a certain extent (Zuckerman et al. 2013). This would reduce the need for religiosity in more intelligent individuals. Third, whilst religiosity can strengthen bonds between people sharing similar views and promote sociability (Norenzayan and Shariff 2008), intelligence is negatively correlated with conformity (Rhodes and Wood 1992), which may be attributable to more intelligent people being less likely to adopt belief systems from their surroundings.

### 1.2. Effect-Strength Differentiation

Although the empirical evidence for a negative association between religiosity and intelligence is overwhelming (see Zuckerman et al. 2020 for an overview), causes of unobserved between-study variability in terms of effect strength (e.g., Dürlinger and Pietschnig 2022) have not yet been conclusively clarified. In particular, different religiosity and cognitive measurement types have been suspected to play a role in the accuracy of effect size estimations.

For instance, associations between religiosity and intelligence are typically less pronounced in studies assessing proxies of intelligence, like Grade Point Average (GPA), in contrast to those with psychometric assessments of intelligence. This is most likely due to school grades being a rather noisy measure of intelligence (Borghans et al. 2016). In addition, studies differ widely with regard to the (psychometric) operationalization of intelligence. For instance, religious beliefs have been shown to correlate more substantially with the performance on matrix tests (*r* = −0.26; Čavojová et al. 2019) than with the performance on vocabulary tests (*r* = −0.12; Kanazawa 2010).

Likewise, correlations between religiosity and intelligence are smaller in strength when religious involvement (like going to church or group membership), as opposed to self-reported religious beliefs, is assessed. This has been assumed to be due to religious involvement being motivated by factors other than religiosity, like social involvement or acceptance (Allport and Ross 1967). Therefore, they can be considered to represent a less salient indicator of the religious beliefs of an individual than self-reported religious beliefs. Considerable variations in the operationalization of religious beliefs could have further contributed to the observed heterogeneity of reported effect sizes between studies.

In some cases, associations with intelligence have been shown to be more pronounced with measures of religious beliefs comprising a single item (*r* = −0.24; Stanovich and West 2007) or just a few items (*r* = −0.12; Kanazawa 2010) compared to validated instruments (*r* = −0.07; Łowicki et al. 2020) like the Centrality of Religiosity Scale (Huber and Huber 2012).

Large-scale cohort-studies may, thus, be useful to assess the stability of the cross-temporal religiosity and intelligence link.

### 1.3. Spirituality and Intelligence

While institutionalized religiosity is on a decline in many Western countries, such as the United States, the number of people describing themselves as spiritual, but not religious, has been increasing (Lipka and Gecewicz 2017). In 2012, about 65% of the adult population in the US described themselves as religious (either in addition to being spiritual or not), and only approximately 18% indicated that they were spiritual but not religious. A mere five years later, in 2017, about a quarter of US adults said they think of themselves as spiritual but not religious, representing an eight-percentage-point increase in five years, whilst only 54% of US-American adults described themselves as religious, representing a decrease of eleven percent.

In contrast to the extensive literature about religiosity and intelligence associations, relationships of spirituality with intelligence have been comparatively little investigated. To the best of our knowledge, only three studies have so far reported associations between spirituality and intelligence, either reporting effect size strengths (*r*s = −0.30 to −0.27; Clark 2004) that resemble the ones established in previous meta-analytical examinations for religiosity and intelligence (Dürlinger and Pietschnig 2022), some “of somewhat lower magnitude”, but “nonetheless consistent with earlier work” (*r* = −0.11; Lace and Evans 2021, p. 4525), or trivial effects (*r* = −0.05; Lewis et al. 2011).

It is difficult to clearly distinguish between the “overlapping circles” (Underwood 2011, p. 30) of religiosity and spirituality. However, they undoubtedly differ in the degree of their formalization. While religiosity is based on an institutionalized system of beliefs, attitudes, and rituals and has been typically assumed to comprise important social and societal aspects, spirituality “involves a personal quest for meaning in life” (Arrey et al. 2016, p. 2).

As a consequence, explanatory mechanisms of negative relationships between religiosity and intelligence cannot be assumed to be identical to those for potential associations between spirituality and intelligence.

On one hand, spirituality might be functionally as equivalent, to a certain extent, with religiosity and with intelligence. For instance, it has been shown that spirituality can have beneficial effects on mental health, which is mediated by enhanced self-control (Shroff et al. 2023) or reduced feelings of loneliness (Gallegos and Segrin 2019) and can empower patients to have a sense of control (Thaker et al. 2006). Moreover, the preference for an analytic cognitive style is negatively associated with reporting embracing a spiritual epistemology (Browne et al. 2014).

On the other hand, a smaller likelihood of more intelligent individuals conforming to religious dogma should not play a decisive role in being spiritual or not. Consequently, it may be reasonable to assume negative associations between spirituality and intelligence but less pronounced associations of spirituality than religiosity with intelligence.

### 1.4. The Present Study

Here, we examine associations between different religiosity and spirituality assessments and crystallized intelligence in the General Social Survey data (GSS), a large representative cohort-based survey of US-American citizens. We assess whether there are associations between (i) religiosity and crystallized intelligence and (ii) spirituality and crystallized intelligence, as well as (iii) whether they generalize over different age groups, cohorts, and religiosity measurement modalities. We, therefore, initially provide direct comparisons between the effect size strength of religiosity and crystallized intelligence associations with spirituality and crystallized intelligence associations for various times of data assessment. Moreover, we assess if the respective correlations change in magnitude over time.

Our study protocol, including all hypotheses and planned confirmatory analyses, was preregistered prior to all data analyses on the Open Science Framework at https://osf.io/jwsd6 on 22 October 2023 (see Supplementary S1 at https://osf.io/k4nqc for deviations from the preregistered protocol).

### 1.5. Hypotheses

First, we expected religiosity and spirituality to be positively correlated because of conceptual overlaps. Second, we hypothesized that religious and spiritual beliefs are both negatively associated with crystallized intelligence. Negative associations of spirituality and crystallized intelligence were expected because we considered some explanatory mechanisms for negative religiosity and intelligence associations to be valid for potential spirituality and intelligence associations as well. We expected these associations to generalize across age groups within, as well as across, cohorts. Third, we expected correlations between religious involvement and crystallized intelligence to be less pronounced than correlations between religious beliefs and crystallized intelligence. However, during times of declining institutionalized religiosity, ongoing participation in religious organizations, religious gatherings, and similar events may conceivably function as a stronger indicator for the actual beliefs of a person than in more religious societies. This assumption is reasonable because social pressure to attend formal religious events can be expected to be lower in less religious societies. Consequently, in this case, attendance can be considered to be a more genuine expression of actual beliefs instead of a social obligation. Therefore, we hypothesized that associations between religious involvement and crystallized intelligence have become more pronounced in more recent years.

## 2. Materials and Methods

### 2.1. Sample

We tested our hypotheses based on US-American individual-level data from 14 cohorts over 34 years (1988–2022) via the General Social Survey (GSS; https://gss.norc.org, accessed on 2 May 2023). The GSS is a population representative cohort-based survey of noninstitutionalized US adults that has been conducted annually or biannually since 1972. Between-cohort data are independent because no participant takes part more than once. We included information from 14 cohorts: 1988, 1991, 1993, 1994, 1998, 2000, 2006, 2008, 2010, 2012, 2014, 2016, 2018, and 2022. Participants’ (*N* = 35,093) age averaged at 47.39 years (*SD* = 17.6), and recruited samples were balanced in terms of sex (19,588 women; 56%; for cohort characteristics, see Table 1).

### 2.2. Measures

#### 2.2.1. Cognitive Abilities

The GSS data include an assessment of vocabulary knowledge that has been administered at most data collection times, namely the WORDSUM vocabulary test. In this ten-item, single-choice task, respondents are asked to find a synonym out of five response options for each of the ten presented stimulus words. Correct responses are awarded one point, thus yielding possible scores ranging from 0 to 10. Verbal ability assessments are education-dependent (Izzaty and Setiawati 2019), thus representing a measure of crystallized intelligence (Kan et al. 2011). Because such tests have been shown to exhibit substantial *g*-loadings (Meijer and Oostdam 2001) as correlations with full-scale IQ reach up to *r* = 0.93 (see Woodley et al. 2015), they can be used as a proxy measure for general intelligence. The WORDSUM vocabulary test has been used in several widely cited studies as an intelligence measure (Caplan and Miller 2010; see also Cor et al. 2012).

#### 2.2.2. Religiosity

Religiosity and spirituality were assessed via two questions in nine cohorts (1998, 2006, 2008, 2010, 2012, 2014, 2016, 2018, 2022): “To what extent do you consider yourself a religious person? Are you…” (not religious at all/slightly religious/moderately religious/very religious) and “To what extent do you consider yourself a spiritual person? Are you…” (not spiritual at all/slightly spiritual/moderately spiritual/very spiritual). Items of this type are among the most valid items to assess religious beliefs (Huber and Huber 2012) and may be, thus, expected to measure spirituality similarly well.

#### 2.2.3. Religious Beliefs

Religious beliefs were assessed by means of a single item in 14 cohorts (1988, 1991, 1993, 1994, 1998, 2000, 2006, 2008, 2010, 2012, 2014, 2016, 2018, 2022): “Please look at this card and tell me which statement comes closest to expressing what you believe about God” (I do not believe in God/I do not know whether there is a God and I do not believe there is any way to find out/I do not believe in a personal God, but I do believe in a Higher Power of some kind/I find myself believing in God some of the time, but not at others/while I have doubts, I feel that I do believe in God/I know God really exists and I have no doubts about it). We only used responses yielding values of 1 (“I don’t believe in God” = not religious) and 6 (“I know God really exists and I have no doubts about it” = religious) in our analyses.

#### 2.2.4. Religious Involvement

In 10 cohorts (1991, 1998, 2006, 2008, 2010, 2012, 2014, 2016, 2018, 2022), religious involvement was assessed via the following question: “How often do you take part in the activities and organizations of a church or place of worship other than attending services?” (never/less than once a year/about once or twice a year/several times a year/about once a month/2–3 times a month/nearly every week/every week/several times a week/once a day/several times a day).

For our main analyses, the original ordinal scaling of our items of self-reported overall religiosity and religious involvement was maintained. We supplemented these calculations with extreme group analyses in our examination of self-reported religiosity and spirituality associations to enhance the power to detect a potential effect. Therefore, we only included religious (very religious) vs. non-religious (not religious at all) and spiritual (very spiritual) vs. non-spiritual individuals (not spiritual at all). Concerning religious involvement, we did not conduct extreme-group comparisons due to the small amount of people who had reported strong involvement. Instead, religious involvement was dichotomized into religiously involved (less than once a year/about once or twice a year/several times a year/about once a month/2–3 times a month/nearly every week/every week/several times a week/once a day/several times a day) vs. not religiously involved individuals (never). All analyses with dichotomized religiosity (religious = very religious/moderately religious/slightly religious vs. not religious = not religious at all) and spirituality (spiritual = very spiritual/moderately spiritual/slightly spiritual vs. not spiritual = not spiritual at all) items are reported in Supplementary S2 at https://osf.io/wpxfy.

### 2.3. Analyses

We used both primary data analyses and meta-analytical approaches to assess cross-sectional associations of religiosity or spirituality with crystallized intelligence, as well as potential cross-temporal changes in effect size strength.

#### 2.3.1. Primary Data Analyses

To examine associations of religious or spiritual beliefs with crystallized intelligence and their potential differences with respect to age groups and cohorts, we conducted multiple linear regressions across all cohorts. Specifically, we regressed scores on the vocabulary test for two dummy-coded variables (religiosity: slightly religious, moderately religious, very religious; reference = not religious at all; spirituality: slightly spiritual, moderately spiritual, very spiritual; reference = not spiritual at all): the age and sex of participants. Moreover, we included interaction terms of age and religiosity, as well as age and spirituality, to assess potential moderations by age. Analogous to our above approach, we repeated these analyses in extreme-group calculations using two dichotomous predictors for religiosity (i.e., very religious vs. not religious at all) and spirituality (i.e., very spiritual vs. not spiritual at all).

Because measures of religious beliefs have been shown to correlate more strongly (negatively) with intelligence than religious involvement (like going to church; Dürlinger and Pietschnig 2022), we examined whether results differed depending on the type of religiosity assessment. In a hierarchical theory-guided stepwise forward regression, we first entered a dichotomous indicator of religious beliefs (I do not believe in God vs. I know God really exists and I have no doubts about it), sex, and participant age to predict crystallized intelligence. We also included an interaction term for religious beliefs and age. In a subsequent step, dummy-coded variables indicating religious involvement (less than once a year, about once or twice a year, several times a year, about once a month, 2–3 times a month, nearly every week, every week, several times a week, once a day, several times a day; reference = never) were added as predictors in the model. Interactions of religious involvement with age were used to assess potential moderating effects of age.

Model fits were compared by examining changes in *R*-squared values. Subsequently, an identical model was calculated with a dichotomous indicator of religious involvement. For all regression models including interaction terms, we first reported results of the respective model with main effects only. We did so to show potential changes in effect size strength after including interactions. In supplementary analyses, we added the year of data collection as a predictor in our stepwise regression to examine potential changes due to cross-temporal effects.

#### 2.3.2. Meta-Analytical Approach

Due to the ordinal scaling of the religiosity and spirituality assessments, we first obtained the Spearman correlation coefficients of (i) religiosity and crystallized intelligence, (ii) spirituality and crystallized intelligence, and (iii) religious involvement and crystallized intelligence within each cohort. In order to investigate time-trends, these precision-weighted (i.e., larger samples being assigned larger weights) coefficients were then meta-regressed on the time of data assessment in three separate models. Analyses were repeated for biserial correlations based on dichotomous indicators of religiosity (very religious vs. not religious at all), spirituality (very spiritual vs. not spiritual at all), and religious involvement (religiously involved vs. not religiously involved).

In addition, we assessed differences in the strength of religiosity with crystallized intelligence and spirituality with crystallized intelligence associations, as well as religious beliefs with crystallized intelligence and religious involvement with crystallized intelligence associations (Myers and Sirois 2006).

Considering the sample sizes at hand, we focused on the interpretation of effect sizes rather than nominal null hypothesis significance testing in our results. We interpret the Pearson correlation coefficients according to Cohen’s well-established classification (Cohen 1988), where absolute *r* = 0.10, 0.30, and 0.50 and *η*^2^ = 0.01, 0.06, and 0.13 values are considered to represent lower thresholds of small, medium, and large effects, respectively (effects smaller than *r* = 0.10 and *η*^2^ = 0.01 are considered to be trivial and not meaningful). Those guidelines have previously been challenged by Gignac and Szodorai (2016), who collated 708 meta-analytically derived (absolute) Pearson correlations and found that only 2.7% were ≥0.50, whereas about 55% were ≤0.21. Consequently, it has been suggested that *r* = 0.10, 0.20 and 0.30 may represent better bottom thresholds for small, medium, and large effects, respectively. All analyses were performed in the open-source software R.4.3.2 using the packages “sjstats” (Lüdecke 2022), “sensemakr” (Cinelli et al. 2021), and “rms” (Harrell 2023). Our entire analytic code is available at https://osf.io/tyb9e.

## 3. Results

### 3.1. Primary Data Analyses

Table 2 shows the Pearson correlations between indicators of religiosity and spirituality across all cohorts. As expected, crystallized intelligence was negatively and non-trivially associated with religiosity and religious beliefs and virtually unrelated to religious involvement. Also in line with our expectations, religiosity and spirituality, as well as religiosity and religious involvement, were positively associated.

In cross-cohort regressions (Table 3), we observed the negative, albeit small, effects of religiosity (assessed with an ordinal response format) on crystallized intelligence. In contrast, spirituality (assessed with an ordinal response format) yielded a positive sign, although effects were trivial in strength. Age showed a small association with crystallized intelligence, thus conforming to the well-known positive age and crystallized IQ link. Interactions with age only reached nominal significance for religiosity, indicating smaller negative effects of religiosity on crystallized intelligence in older ages, although effects were merely trivial. Sex did not yield any meaningful effects on crystallized intelligence.

Regressing crystallized intelligence on extreme groups (very religious vs. not religious at all; very spiritual vs. not spiritual at all) showed a significant negative small effect of religiosity (*β* = −0.945, *p* < 0.001, *η*^2^ = 0.02) and a significant positive but trivial effect of spirituality on crystallized intelligence (*β* = 0.271, *p* < 0.05, *η*^2^ = 0.003). When interactions were included, the main effects changed in terms of signs, although effects did not reach nominal significance (religiosity: *β* = 0.315, *p* = 0.341, *η*^2^ = 0.02; spirituality: *β* = −0.232, *p* = 0.485, *η*^2^ = 0.003). The interaction between age and religiosity was significant (*β* = −0.026, *p* < 0.001, *η*^2^ = 0.006), indicating nominally weaker negative effects of religiosity on crystallized intelligence in older ages, although they were trivial in strength.

Effects of religious beliefs and religious involvement on crystallized intelligence are detailed in Table 4. Religious beliefs were negatively associated with crystallized intelligence in both models with and without indicators of religious involvement. Interestingly, compared with the reference category (never), indicators of religious involvement yielded mostly positive signs, which became nominally significant for seven categories (less than once a year, about once or twice a year, several times a year, about once a month, 2–3 times a month, nearly every week, every week) in the model without interactions, although all of them were trivial in strength. Effect strengths of interaction terms were not meaningful, providing no evidence for moderating effects of participants’ age. Although Model 2 (*R*^2^ = 0.031) explained significantly (*F* = 4.505, *p* < 0.001) more variance than Model 1 (*R*^2^ = 0.023), the effects of the added variables were merely trivial, thus indicating beliefs as the strongest predictor for crystallized intelligence. Variance inflation factors yielded no evidence for multicollinearity (all *VIF*s < 1.23 in models without interactions).

Originally, we had preregistered these analyses with an inverse order of included predictors (i.e., we first intended to include indicators of religious involvement and, subsequently, indicators of religious beliefs) and consequently provide corresponding results in Supplementary S3 (https://osf.io/fkjdr), which were in line with the findings of our main analyses.

Table 5 shows the effects of dichotomized religious involvement on crystallized intelligence. Again, religious beliefs had a negative impact on crystallized intelligence, whereas religious involvement had a positive effect. After including religious involvement, the adjusted *R*^2^ increased significantly from 0.023 to 0.029 (*F* = 24.261; *p* < 0.001), but again the effects of religious involvement were trivial and beliefs were the strongest predictor for crystallized intelligence.

The data collection year did not show any significant influences when it was added as a predictor in our supplementary analyses of both dummy-coded and dichotomized religious involvement. The meaningfulness of the other predictors remained virtually unchanged, thus suggesting that our results were generalized over data collection years (for numerical detail, see Supplementary S4 at https://osf.io/9crp5).

### 3.2. Meta-Analytical Results

In line with the results of our stepwise regressions, cohort-specific correlations between religiosity and crystallized intelligence were consistently negative, whereas associations between spirituality and crystallized intelligence showed positive signs but trivial correlation strengths in all cohorts (left columns in Table 6). Formal analyses indicated that the strength of the religiosity and crystallized intelligence association differed significantly from that of the spirituality and crystallized intelligence correlation in all cohorts (all *p*s < 0.01; Table 6). Correlations of religiosity and crystallized intelligence (*ß* = −0.007, *p* = 0.041, *R*^2^ = 0.451 *η*^2^ = 0.53) were consistently negative but increased in magnitude in more recent years, while those of spirituality and crystallized intelligence (*ß* = −0.006, *p* = 0.042, *R*^2^ = 0.445 *η*^2^ = 0.08) changed signs and became negative in later cohorts, but they remained trivial in terms of effect strength.

We repeated these analyses with extreme groups (very religious vs. not religious at all, very spiritual vs. not spiritual at all), yielding broadly conforming results compared to ordinal analyses. For all cohorts, we found negative correlations of religiosity and crystallized intelligence, whereas spirituality and crystallized intelligence correlations were mostly positive in terms of sign but trivial in terms of strength (rightmost columns in Table 6). Again, within all cohorts, religiosity and crystallized intelligence correlation coefficients differed significantly in terms of strength compared to those of spirituality and crystallized intelligence.

Point-biserial correlations between religiosity and crystallized intelligence (*ß* = −0.004, *p* = 0.213, *R*^2^ = 0.118 *η*^2^ = 0.24) did not change significantly across cohorts, while those of spirituality and crystallized intelligence (*ß* = −0.007, *p* = 0.026, *R*^2^ = 0.520 *η*^2^ = 0.59) decreased across the cohorts. The observed negative associations between religiosity and crystallized intelligence were in line with our expectations; however, in contrast to our expectations, spirituality was virtually unrelated to crystallized intelligence.

We obtained correlation coefficients of religious involvement and crystallized intelligence for each cohort (Table 7). Religious involvement was virtually unrelated to crystallized intelligence. No meaningful significant changes in effect size strengths over time were observed (*ß* = −0.003, *p* = 0.285, *R*^2^ = 0.051, *η*^2^ = 0.19). A similar pattern was observed after dichotomizing religious involvement, showing no meaningful changes in effect strength over time (*ß* = −0.001, *p* = 0.449, *R*^2^ = −0.052, *η*^2^ = 0.10). Associations of religious beliefs with crystallized intelligence were consistently negative and mostly non-trivial in terms of strength. Interestingly, negative associations between religious beliefs and crystallized intelligence increased in strength across cohorts, yielding a strong effect (*ß* = −0.008, *p* = 0.005, *R*^2^ = 0.711 *η*^2^ = 0.75). Our findings supported our hypothesis of less pronounced correlations of religious involvement with crystallized intelligence compared to correlations of religious beliefs with crystallized intelligence. However, we did not observe any changes in the effect strengths of religious involvement and crystallized intelligence associations over time.

## 4. Discussion

Here, we provide evidence for religiosity and crystallized intelligence, as well as spirituality and crystallized intelligence, associations in 14 population-representative US-based cohorts from 1988 to 2022. We show that crystallized intelligence is negatively related to religiosity but unrelated to spirituality. Our results present several points of interest, as discussed below.

First, as expected, we found small but meaningful negative associations of religious beliefs with crystallized intelligence as well as negative associations of self-reported religiosity with crystallized intelligence. We found tentative evidence for less pronounced associations between self-reported religiosity and crystallized intelligence associations in older ages. These findings are consistent with the idea of protective effects of religiosity on age-related cognitive decline, which have been demonstrated for American samples (Corsentino et al. 2009; Van Ness and Kasl 2003), but failed to be replicated in (Western) Europe (Dürlinger et al. 2023; Ritchie et al. 2014). Such effects were mainly attributed to religiosity leading to an increase in activities that are likely to stimulate cognitive functions (such as praying, studying scriptural texts, or mere general socializing; Hill 2008).

Interestingly, associations in earlier cohorts showed significantly smaller associations between religiosity and crystallized intelligence compared to subsequent cohorts, thus indicating cross-temporally increasing effect size strengths. When explicitly examining associations between belief in God and crystallized intelligence, we also found an increase in effect size strength across cohorts, but no effects of age. This pattern suggests stronger influences of social changes over time than of participant ages on religiosity and crystallized intelligence associations. It may be speculated that this may be due to individuals having become more comfortable with describing themselves as not being religious due to society having become less conservative and more permissive. Conceivably, an increasing polarization of the political landscape in the USA could be another reason that may drive individuals to express their religiosity as an expression of party affiliation (Margolis 2018).

Second, religious involvement was unrelated to crystallized intelligence in our data. Less pronounced associations of crystallized intelligence with religious involvement than with religious beliefs were to be expected and are in line with previous findings (Dürlinger and Pietschnig 2022; Zuckerman et al. 2013), although the presently observed virtually nil effect was somewhat unexpected. Lower associations are expectable because taking part in a religious organization or ceremony constitutes a weaker indicator for the actual beliefs of a person than self-reports of being religious. The virtual null associations of religious involvement and crystallized intelligence, however, are noteworthy and may indicate that religious involvement might not be motivated by the same causes as personal beliefs.

We did not observe any effects of age regarding religious involvement. Moreover, in contrast to associations between religious beliefs and crystallized intelligence, associations of religious involvement with crystallized intelligence did not change across cohorts. It is reasonable to expect cross-temporal changes in the involvement and crystallized intelligence association only, because when institutionalized religiosity becomes less prevalent in a society, being part of a religious organization may be considered to represent a better indicator for the actual beliefs of a person. This can be attributed to attendance at religious events being likely a more genuine expression of actual beliefs instead of a social obligation in less religious societies. In more religious societies, attendance in religious events and organizations may often be a consequence of extrinsic motivators such as societal pressure. This is also indicated by a decline in religious involvement in societies becoming more liberal and pluralistic with regard to religiosity (Fox and Tabory 2008; Froese and Pfaff 2001). The effect-strength increases in religiosity and crystallized intelligence associations across different cohorts seem to be consistent with smaller negative associations between self-reported religiosity and crystallized intelligence in older participants. Both might be attributed to a decreasing societal value of religiosity in the US-American population (Lipka and Gecewicz 2017).

Finally, we did not observe any meaningful associations of self-reported spirituality with crystallized intelligence. These findings contrast previous reports of negative spirituality and intelligence associations (Clark 2004; Lace and Evans 2021). Although we expected associations with crystallized intelligence to be less pronounced for spirituality than for religiosity, it seems surprising that spirituality was practically unrelated to crystallized intelligence. The conceptual overlaps between the religiosity and spiritually constructs may lead researchers to expect that well-established correlational patterns like those of religiosity with intelligence may generalize, to a certain extent, to spirituality associations. Whilst our findings may support the idea that religiosity and intelligence could be functionally equivalent to a certain extent, spirituality and intelligence do not appear to be functionally overlapping at all.

This is further supported by evidence for systematic differences between religious and spiritual individuals. For instance, it has been shown that high openness values predict spirituality positively but religious fundamentalism negatively (Saroglou 2010). Considering the well-established positive openness to experience with intelligence association (especially with crystallized domains, for meta-analytic review, see Anglim et al. 2022; Judge et al. 2007, etc.), these results appear to be in line with our findings.

Importantly, it needs to be emphasized that the presently observed negative associations between IQ and religious beliefs indicate effects at the population level. We do not mean to suggest that, on an individual level, religious believers are necessarily less intelligent than non-believers.

### Limitations

In terms of limitations, it needs to be acknowledged that in our archival data analyses, religiosity and spirituality were each assessed with a single item. Single items have sometimes been criticized due to their inferior or sometimes unknown reliability and validity compared to multi-item scales (Allen et al. 2022). However, it has been demonstrated in the context of educational research that single items may provide useful and psychometrically sound alternatives when scale scores are unavailable (Gogol et al. 2014). Moreover, the strength of the presently observed religiosity and crystallized intelligence link closely resembles those from earlier accounts that used self-report scales to assess religiosity (Lewis et al. 2011; Łowicki et al. 2020), thus providing indirect evidence of the reliability and validity of our items. In fact, religiosity has repeatedly been assessed by means of single items (e.g., Daws and Hampshire 2017; Drewelies et al. 2018; Furnham and Grover 2020; Hartman et al. 2017; Razmyar and Reeve 2013; Shenhav et al. 2011; Sherkat 2010, 2011).

Single items of religiosity have been shown to be good indicators of intrinsic proreligious positions (Gorsuch and McFarland 1972), and recent findings demonstrate the usefulness and predictive validity of such assessments in general (Song et al. 2023). The usefulness of single items in the context of religiosity is further supported by the short form of the Centrality of Religiosity Scale, the CRS-5 (Huber and Huber 2012). The CRS-5 comprises five single-item religiosity subscales such as public practice (“How often do you take part in religious services?”) or ideology (“To what extent do you believe that God or something divine exists?”). In a recent study, both of these items have been shown to yield the strongest factor loadings on personal religiosity (Ackert and Plopeanu 2021), thus demonstrably representing valid religiosity assessments. Because these two items had been administered in the GSS, we used them as appropriate indicators of religiosity in the present analyses.

In addition, relatively few people have reported that they do not belief in God. In most cohorts, around 60 participants, and in 1988, even as few as 22, chose this answer option. We only conducted extreme group comparisons here because we considered other answer options to be too vague to conduct meaningful analyses. Because this low case number could have underestimated the strength of correlations with other variables, reported effect sizes may be seen as the lower threshold of true effect strengths. The consistency of our results in terms of effect strength and size, however, support our interpretations.

## 5. Conclusions

In conclusion, we show here negative associations of religiosity but none for spirituality with crystallized intelligence in fourteen population-representative cohorts of US citizens from 1988 to 2022. These results were broadly generalized across age groups, cohorts, and analytical approaches, thus suggesting that religiosity and intelligence may possibly be functionally equivalent to some extent, whilst spirituality represents a distinct construct that is not. Increasing associations of religious beliefs with crystallized intelligence across cohorts may conceivably be attributed to the decreasing societal value of religiosity in the USA.

## Figures and Tables

**Table 1:** Participant characteristics according to cohort.

Year	1988	1991	1993	1994	1998	2000	2006	2008	2010	2012	2014	2016	2018	2022
*N*	1481	1517	1606	2992	2832	2817	4510	2023	2044	1974	2538	2867	2348	3544
Percentage of men within samples	0.431	0.419	0.427	0.431	0.435	0.436	0.444	0.46	0.436	0.448	0.45	0.445	0.448	0.462
Age Mean	45.37	45.63	46.05	45.97	45.56	46.02	47.14	47.71	47.97	48.19	49.01	49.16	48.97	49.18
Age SD	18.32	17.81	17.36	17.05	17.10	17.37	16.89	17.35	17.68	17.69	17.41	17.69	18.06	17.97
I do not believe in God	22 (1.5)	31 (2.3)	44 (2.9)	33 (2.5)	40 (3.2)	33 (2.8)	66 (2.2)	60 (3.0)	66 (3.3)	65 (3.3)	87 (3.5)	126 (4.4)	109 (4.7)	158 (6.8)
I know God really exists and I have no doubts about it	939 (63.5)	834 (62.8)	992 (66.3)	860 (64.9)	775 (62.8)	766 (65.8)	1876 (63.3)	1240 (61.8)	1181 (58.4)	1168 (59.7)	1468 (58.2)	1607 (56.6)	1262 (54.5)	1159 (49.6)
Religious involvement														
Never	.	507 (38.1)	.	.	471 (38.0)	.	1,325 (4.5)	861 (42.9)	864 (42.5)	887 (45.3)	1139 (45.1)	1286 (45.2)	1087 (46.9)	1635 (46.8)
Less than once a year	.	160 (12.0)	.	.	147 (11.9)	.	249 (8.4)	148 (7.4)	164 (8.1)	139 (7.1)	197 (7.8)	187 (6.6)	148 (06.4)	413 (11.8)
About once or twice a year	.	171 (12.9)	.	.	136 (11.0)	.	368 (12.4)	300 (15.0)	292 (14.4)	251 (12.8)	353 (14.0)	384 (13.5)	304 (13.1)	438 (12.6)
Several times a year	.	128 (9.6)	.	.	131 (10.6)	.	290 (9.7)	270 (13.5)	238 (11.7)	183 (9.3)	261 (10.3)	327 (11.5)	241 (10.4)	365 (10.5)
About once a month	.	83 (6.2)	.	.	64 (5.2)	.	215 (7.2)	105 (5.2)	120 (5.9)	118 (6.0)	146 (5.8)	183 (6.4)	154 (6.6)	164 (4.7)
2–3 times a month	.	82 (6.2)	.	.	66 (5.3)	.	174 (5.8)	108 (5.4)	140 (6.9)	129 (6.6)	140 (5.5)	162 (5.7)	145 (6.3)	145 (4.2)
Nearly every week	.	61 (4.6)	.	.	54 (4.4)	.	88 (3.0)	43 (2.1)	50 (2.5)	64 (3.3)	76 (3.0)	69 (2.4)	37 (1.6)	85 (2.4)
Every week	.	89 (6.7)	.	.	88 (7.1)	.	236 (7.9)	143 (7.1)	137 (6.7)	167 (8.5)	183 (7.3)	213 (7.5)	180 (7.8)	207 (5.9)
Several times a week	.	48 (3.6)	.	.	66 (5.3)	.	17 (0.6)	13 (0.6)	21 (1.0)	9 (0.5)	21 (0.8)	21 (0.7)	13 (0.6)	10 (0.3)
Once a day	.	.	.	.	8 (0.6)	.	17 (0.6)	15 (0.7)	5 (0.2)	12 (0.6)	8 (0.3)	16 (0.6)	9 (0.4)	28 (0.8)
Several times a day	.	.	.	.	8 (0.6)	.	.	.	.	.	.	.	.	.
Self-reported religiosity														
Not religious at all	.	.	.		218 (15.3)	.	444 (14.9)	317 (15.8)	375 (18.5)	382 (19.6)	519 (20.6)	631 (22.3)	510 (21.9)	1051 (30.0)
Slightly religious	.	.	.		335 (23.5)	.	669 (22.5)	466 (23.3)	470 (23.2)	426 (21.8)	609 (24.2)	647 (22.8)	573 (24.6)	841 (24.0)
Moderately religious	.	.	.		606 (42.5)	.	1285 (43.3)	843 (42.2)	842 (41.5)	784 (40.2)	958 (38.0)	1091 (38.5)	873 (37.5)	1124 (32.1)
Very religious	.	.	.		268 (18.8)	.	572 (19.3)	374 (18.7)	343 (16.9)	360 (18.4)	434 (17.2)	464 (16.4)	372 (16.0)	488 (13.9)
Self-reported spirituality														
Not spiritual at all	.	.	.		171 (12.0)	.	266 (09.0)	196 (09.8)	219 (10.8)	203 (10.5)	268 (10.7)	284 (10.0)	265 (11.4)	502 (14.4)
Slightly spiritual	.	.	.		366 (25.7)	.	595 (20.1)	429 (21.5)	433 (21.4)	412 (21.4)	578 (23.0)	645 (22.8)	522 (22.5)	831 (23.8)
Moderately spiritual	.	.	.		571 (40.2)	.	1216 (41.2)	817 (41.0)	787 (38.9)	731 (37.9)	935 (37.2)	1068 (37.7)	831 (35.8)	1202 (34.4)
Very spiritual	.	.	.		314 (22.1)	.	877 (29.7)	549 (27.6)	583 (28.8)	583 (30.2)	735 (29.2)	833 (29.4)	700 (30.2)	957 (27.4)

Note. Cell entries represent absolute numbers of observations unless indicated otherwise in line labels; numbers in parentheses represent percentages. For belief in God, only two response options were used—percentages relate to all given answers.

**Table 2:** Correlations between religiosity and spirituality indicators across all cohorts.

	Crystallized Intelligence	Religiosity	Spirituality	Religious Beliefs	Religious Involvement	Age
Crystallized intelligence	-					
Religiosity	−0.130 ***	-				
Spirituality	0.032 ***	0.570 ***	-			
Religious beliefs	−0.106 ***	0.394 ***	0.328 ***	-		
Religious involvement	0.003	0.499 ***	0.398 ***	0.238 ***	-	
Age	0.075 ***	0.208 ***	0.141 ***	0.058 ***	0.102 **	-
Sex	0.019 **	0.120 ***	0.173 ***	0.130 ***	0.082 ***	0.025 ***

Note. Cell entries are correlation coefficients for the total sample. Correlations with the ordinal response format were originally calculated as Spearman coefficients and then transformed into Pearson coefficients. Sex: 0 = men; 1 = women. **: *p* < .01; ***: *p* < .001.

**Table 3:** Regression over all cohorts of crystallized intelligence on religiosity and spirituality.

	Without Interactions			With Interactions		
	*β*	*SE*	*η* ^2^	*β*	*SE*	*η* ^2^
Slightly religious	−0.870 ***	0.057	0.020	−0.377 *	0.161	0.006
Moderately religious	−1.101 ***	0.055	0.006	−0.261	0.158	0.006
Very religious	−1.384 ***	0.069	0.003	−0.351	0.209	0.003
Slightly spiritual	0.214 **	0.070	0.001	0.002	0.183	0.001
Moderately spiritual	0.541 ***	0.069	0.007	0.147	0.187	0.007
Very spiritual	0.698 ***	0.073	0.004	0.618 **	0.203	0.004
Age	0.015 ***	0.001	0.020	0.024 ***	0.003	0.020
Sex (0 = men, 1 = women)	0.071	0.037	<0.001	0.077 *	0.037	<0.001
Slightly religious * age				−0.012 ***	0.003	0.001
Moderately religious * age				−0.019 ***	0.003	0.002
Very religious * age				−0.022 ***	0.004	0.001
Slightly spiritual * age				0.005	0.004	<0.001
Moderately spiritual * age				0.008 *	0.004	<0.001
Very spiritual * age				0.002	0.004	<0.001

Note. In the leftmost columns, we report results without interaction terms. In the rightmost columns, interactions between religiosity or spirituality and age are added. Religiosity and spirituality are dummy-coded with the reference categories “not religious at all” and “not spiritual at all”; all variance inflation factors (*VIF*s) < 3.4. * = *p* < .05; ** = *p* < .01; *** = *p* < .001.

**Table 4:** Stepwise regressions of crystallized intelligence’s impact on religious beliefs and dummy-coded religious involvement.

	Without Interactions						With Interactions					
	Model 1			Model 2			Model 1			Model 2		
	*β*	*SE*	*η* ^2^	*β*	*SE*	*η* ^2^	*β*	*SE*	*η* ^2^	*β*	*SE*	*η* ^2^
Belief	−1.126 ***	0.098	0.020	−1.278 ***	0.100	0.020	−0.894 ***	0.264	0.020	−1.111 ***	0.272	0.020
Age	0.010 ***	0.001	0.007	0.009 ***	0.001	0.008	0.015 **	0.005	0.007	0.014 **	0.005	0.008
Sex	0.141 **	0.047	0.001	0.126 **	0.047	0.001	0.141 **	0.047	0.001	0.126 **	0.047	0.001
Belief * age							−0.005	0.005	<0.001	−0.004	0.006	<0.001
Less than once a year				0.192 *	0.096	<0.001				0.016	0.286	<0.001
About once or twice a year				0.226 **	0.076	<0.001				0.248	0.225	<0.001
Several times a year				0.406 ***	0.074	0.001				0.563 *	0.230	0.001
About once a month				0.224 *	0.091	<0.001				−0.051	0.266	<0.001
2–3 times a month				0.361 ***	0.090	0.001				0.782 **	0.283	0.001
Nearly every week				0.644 ***	0.125	0.002				0.773	0.395	0.002
Every week				0.491 ***	0.081	0.005				0.854 ***	0.253	0.005
Several times a week				0.244	0.211	<0.001				1.117	0.711	<0.001
Once a day				0.093	0.290	<0.001				1.544	1.077	<0.001
Less than once a year * age										0.004	0.006	<0.001
About once or twice a year * age										<−0.001	0.005	<0.001
Several times a year * age										−0.003	0.004	<0.001
About once a month * age										0.005	0.005	<0.001
2–3 times a month * age										−0.008	0.005	<0.001
Nearly every week * age										−0.003	0.007	<0.001
Every week * age										−0.007	0.005	<0.001
Several times a week * age										−0.018	0.014	<0.001
Once a day * age										−0.028	0.020	<0.001

Note. In the leftmost columns, we report results without interaction terms. In the rightmost columns, interactions between religious beliefs or involvement and age were added. In Model 1, crystallized intelligence was regressed on an indicator of religious beliefs. In Model 2, dummy-coded indicators of religious involvement were added. The “several times a day” category was omitted due to low case numbers (*n* = 8). Sex: 0 = men; 1 = women. Religious beliefs: 0 = “I don’t believe in God”; 1 = “I know God really exists and I have no doubts about it”. All variance inflation factors (*VIF*s) < 1.3. * = *p* < .05, ** = *p* < .01, and *** = *p* < .001.

**Table 5:** Stepwise regressions of crystallized intelligence’s impact on religious beliefs and religious involvement (yes/no).

	Without Interaction						With Interactions					
	Model 1			Model 2			Model 1			Model 2		
	*β*	*SE*	*η* ^2^	*β*	*SE*	*η* ^2^	*β*	*SE*	*η* ^2^	*β*	*SE*	*η* ^2^
Religious beliefs	−1.126 ***	0.098	0.020	−1.270 ***	0.100	0.020	−0.894 ***	0.264	0.020	−1.091 ***	0.271	0.020
Age	0.010 ***	0.001	0.007	0.010 ***	0.001	0.008	0.015 **	0.005	0.007	0.014 **	0.005	0.008
Sex	0.141 **	0.047	0.001	0.131 **	0.047	0.001	0.141 **	0.047	0.001	0.130 **	0.047	0.001
Religious beliefs * age							−0.005	0.005	<0.001	−0.004	0.006	<0.001
Religious involvement				0.341 ***	0.049	.007				0.412 **	0.146	0.007
Religious involvement * age										−0.002	0.003	<0.001

Note. Sex: 0 = men; 1 = women. Religious beliefs: 0 = “I don’t believe in God”; 1 = “I know God really exists and I have no doubts about it”. All variance inflation factors (*VIF*s) < 1.1. * = *p* < .05, ** = *p* < .01, and *** = *p* < .001.

**Table 6:** Correlations between religiosity or spirituality and intelligence.

Cohort	Religiosity and Crystallized Intelligence (*n*)	Spirituality and Crystallized Intelligence (*n*)	*z*_1_	Extreme Groups: Religiosity and Crystallized Intelligence (*n*)	Extreme Groups: Spirituality and Crystallized Intelligence (*n*)	*z*_2_
2006	−0.046 (1382)	0.071 * (1376)	−3.062 **	−0.147 ** (477)	0.065 * (532)	−3.370 ***
2008	−0.035 (1150)	0.085 ** (1149)	−2.893 **	−0.113 * (403)	0.091 (435)	−2.943 **
2010	−0.166 *** (1380)	0.030 (1375)	−5.181 ***	−0.260 *** (492)	0.002 (562)	−4.339 ***
2012	−0.123 *** (1264)	0.055 (1252)	−4.486 ***	−0.201 *** (479)	0.031 (495)	−3.653 ***
2014	−0.093 *** (1648)	0.060 * (1646)	−4.400 ***	−0.184 *** (608)	0.040 (653)	−3.998 ***
2016	−0.154 *** (1845)	0.018 (1842)	−5.269 ***	−0.219 *** (718)	−0.006 (718)	−4.101 ***
2018	−0.105 *** (1533)	0.075 ** (1530)	−4.970 ***	−0.153 *** (569)	0.048 (651)	−3.515 ***
2022	−0.189 *** (2279)	−0.043 (2276)	−5.015 ***	−0.241 *** (1003)	−0.067 * (967)	−3.962 ***

Note. All correlations are Pearson coefficients. In the leftmost columns, correlations between religiosity or spirituality and crystallized intelligence are provided. Pearson coefficients allow for formal comparisons of correlation coefficient strengths. *z*_1_ represents test statistics for such comparisons between religiosity or spirituality and crystallized intelligence associations. Point-biserial correlations from extreme groups (very religious vs. not religious at all, very spiritual vs. not spiritual at all) are reported in the rightmost columns. *z*_2_ represents test statistics for comparisons of correlation coefficient strengths based on extreme groups. Numbers in parentheses represent the respective sample sizes. *: *p* < .05; **: *p* < .01; ***: *p* < .001.

**Table 7:** Correlations between religious involvement and crystallized intelligence within cohorts.

Year	Religious Involvement and Crystallized Intelligence *r* (*n*)	Religious Beliefs and Crystallized Intelligence *r* (*n*)	*z*_1_	Religious Involvement (Yes/No) and Crystallized Intelligence *r* (*n*)	*z*_2_
2006	0.006 (1387)	−0.042 (911)	1.127	0.011 (1387)	−1.228
2008	0.093 ** (1154)	−0.067 (746)	3.397 ***	0.064 * (1154)	−2.776 **
2010	−0.029 (1380)	−0.135 *** (863)	2.454 **	−0.027 (1380)	−2.496 **
2012	0.008 (1271)	−0.129 *** (792)	3.052 **	0.003 (1271)	−2.938 **
2014	0.038 (1647)	−0.136 *** (1028)	4.380 ***	0.023 (1647)	−4.016 ***
2016	<−0.001 (1856)	−0.128 *** (1117)	3.386 ***	0.012 (1856)	−3.717 ***
2018	0.005 (1525)	−0.131 *** (897)	3.239 **	0.008 (1525)	−3.315 ***
2022	−0.019 (2280)	−0.218 *** (644)	4.528 ***	−0.006 (2280)	4.827 ***

Note. All correlations are Pearson coefficients. Correlations between religious involvement and crystallized intelligence are displayed in the left column. Correlations between religious beliefs and crystallized intelligence are provided in the second column. *z*_1_ corresponds to tests for significant differences in terms of strength of associations between religious involvement and crystallized intelligence with those between religious beliefs and crystallized intelligence. In the fourth column, point-biserial correlations between religious involvement and crystallized intelligence are displayed. *z*_2_ corresponds to tests for significant differences in terms of strength of associations between religious involvement (dichotomized) and crystallized intelligence with those between religious beliefs and crystallized intelligence. Numbers in parentheses represent the respective sample size. *: *p* < .05; **: *p* < .01; ***: *p* < .001.

## Data Availability

We used data from the GSS: https://gss.norc.org/Get-The-Data (obtained May–August 2023). Further modifications are reported in our analytic code, which is available at https://osf.io/rhncm.

## Supplementary Materials

S1 at https://osf.io/k4nqc for deviations from the preregistered protocol; S2 at https://osf.io/wpxfy for analyses with dichotomized religiosity and spirituality items; S3 at https://osf.io/fkjdr for an inverse order of included predictors; S4 at https://osf.io/9crp5 for analyses with data collection year as a predictor. The analytic code is available at https://osf.io/tyb9e.
